# A prototype non-invasive urodynamic test to estimate voiding reserve in normal adult males

**DOI:** 10.1080/2090598X.2019.1649892

**Published:** 2019-08-29

**Authors:** Shafik Shoukry, Mostafa Elmissiry, Ahmed Abulfotooh, Ahmed Moussa, Wally Mahfouz, Waleed Dawood, Aly Abdel-Karim, Mohamed Hassouna

**Affiliations:** Section of Voiding Dysfunction and Urodynamics, Department of Urology, Alexandria University, Alexandria, Egypt

**Keywords:** Voiding, reserve, non-invasive, urodynamics, male

## Abstract

**Objective**: To propose a prototype non-invasive test to estimate voiding reserve in normal adult men; identifying its feasibility, limitations, and initial results.

**Subjects and methods**: In all, 30 adult healthy male volunteers aged <40 years were included in the study. Initial free uroflowmetry was done with post-void residual urine volume (PVR) assessment using ultrasonography. The men were later asked to void into a uroflowmeter through a condom catheter attached to the glans penis and connected to an outflow tube with specific vertical heights (10, 20, 30, 40, 50 and 60 cm) on different days. The mean maximum urinary flow rate (Q_max_) and PVR at each height were compared with the Q_max_ and PVR at the initial free uroflowmetry. The maximum height at which the Q_max_ and PVR remained normal was considered the normal voiding reserve for that age group.

**Results**: All the men completed the study without any complications. At zero level, the mean Q_max_ was 27.6 mL/s, which then dropped gradually to reach 17.8 mL/s at 60 cm, where still 83% of the men had a normal Q_max_. The PVR was nil at zero level and started to exceed the normal range at 50 and 60 cm height (58 and 65.7 mL, respectively). So, the maximum height resistance at which the men could have a normal Q_max_ and normal PVR was 40 cm.

**Conclusions**: The use of the tube height-resistance test to assess voiding reserve is feasible, non-invasive and has no complications. A 40-cm height resistance can be considered a reference level that a young adult male should be tested against to estimate his voiding reserve.

**Abbreviations**: NPV: negative predictive value; P_det_Q_max_: maximum detrusor pressure at maximum urinary flow; PPV: positive predictive value; PVR: post-void residual urine volume; ROC: receiver operating characteristic

## Introduction

The lower urinary tract has a reserve that allows it to overcome increasing outlet resistance. It plays an important role in patients with BOO. The detrusor muscle can compensate for increased outlet resistance to a certain point after which bladder decompensation occurs [1,2]. Prolonged untreated BOO leads to impaired detrusor contractility, which manifests as chronic urinary retention and abnormally high post-void residual urine volume (PVR) [3]. Identifying the limits of the voiding reserve that can overcome the outflow resistance would be a very beneficial tool in the hand of urologists.

To date, no generalised consensus regarding the limits of voiding reserve is available in the literature. Yalla and Sullivan [4] described the detrusor reserve as the difference between the projecting isometric pressure and maximum detrusor pressure at maximum urinary flow (P_det_Q_max_) after performing a pressure–flow study twice. However, they did not determine normal values or ranges for detrusor muscle reserve. Moreover, this requires performing a pressure–flow urodynamic test, which is considered an invasive test and is not generally accepted by many patients. Moreover, invasive urodynamic testing carries some possible morbidities including dysuria, bacteriuria, haematuria, and retention [5,6].

Several published studies have tried to test the detrusor function by creation of controlled outflow resistance. However, most of these studies faced severe difficulties, inaccuracy and failure of reproduction of results [7]. Our present trial is an initial effort to quantify voiding reserve using a non-invasive novel urodynamic test.

The basic concept of our novel test is to measure the ability of the lower urinary system to adequately empty the bladder with a Q_max_ and PVR within normal ranges despite rising outflow resistance. We propose an initial prototype of this non-invasive test using a controlled graduated outflow resistance to uroflowmetry to evaluate voiding reserve.

The aim of the present study was to evaluate the feasibility of an initial prototype of a non-invasive urodynamic test to estimate the voiding reserve in normal adult males, identifying its pitfalls and its initial results.

## Subjects and methods

After local Institutional Review Board approval, 30 adult male volunteers aged <40 years were included in the study. We aimed at studying this test in normal young males initially before applying it to our target population of obstructed elderly males. Informed consent was obtained from all participants included in the study. Exclusion criteria were males with known symptomatic or radiological infra-vesical obstruction and/or Q_max_ <15 mL/s or PVR >50 mL.

**Figure 1.**
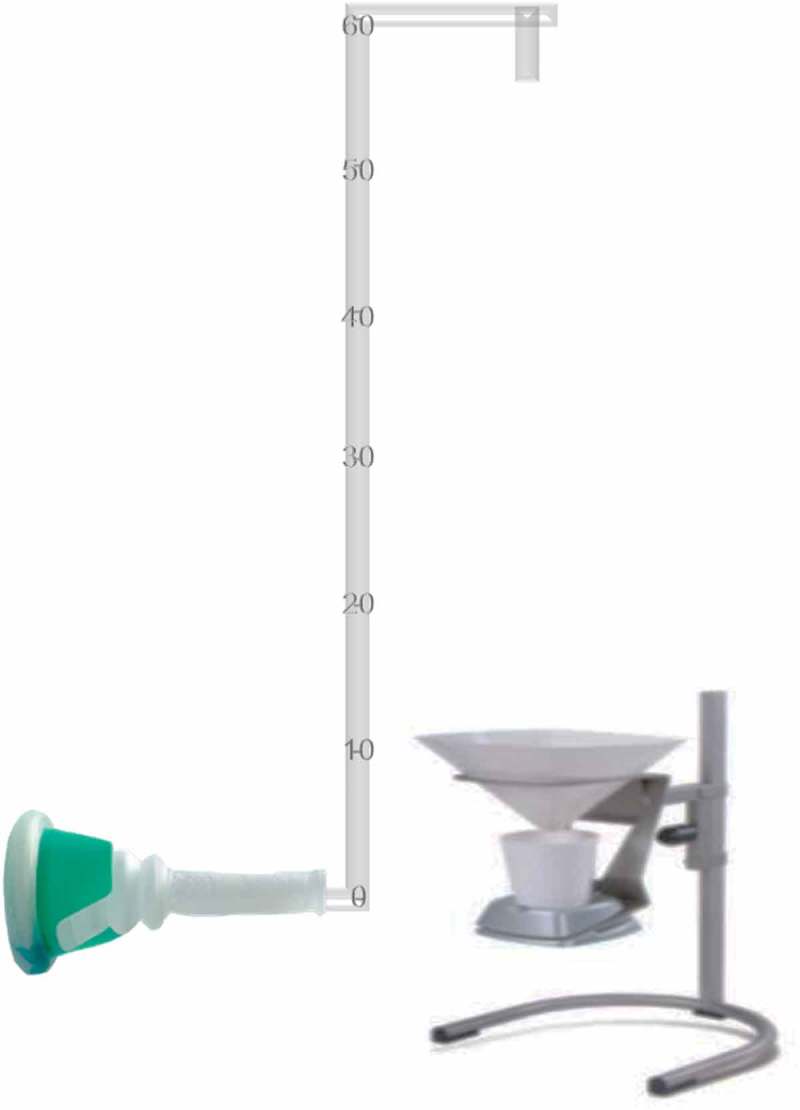
Illustration of the non-invasive tube height resistance test for voiding reserve.

**Figure 2.**
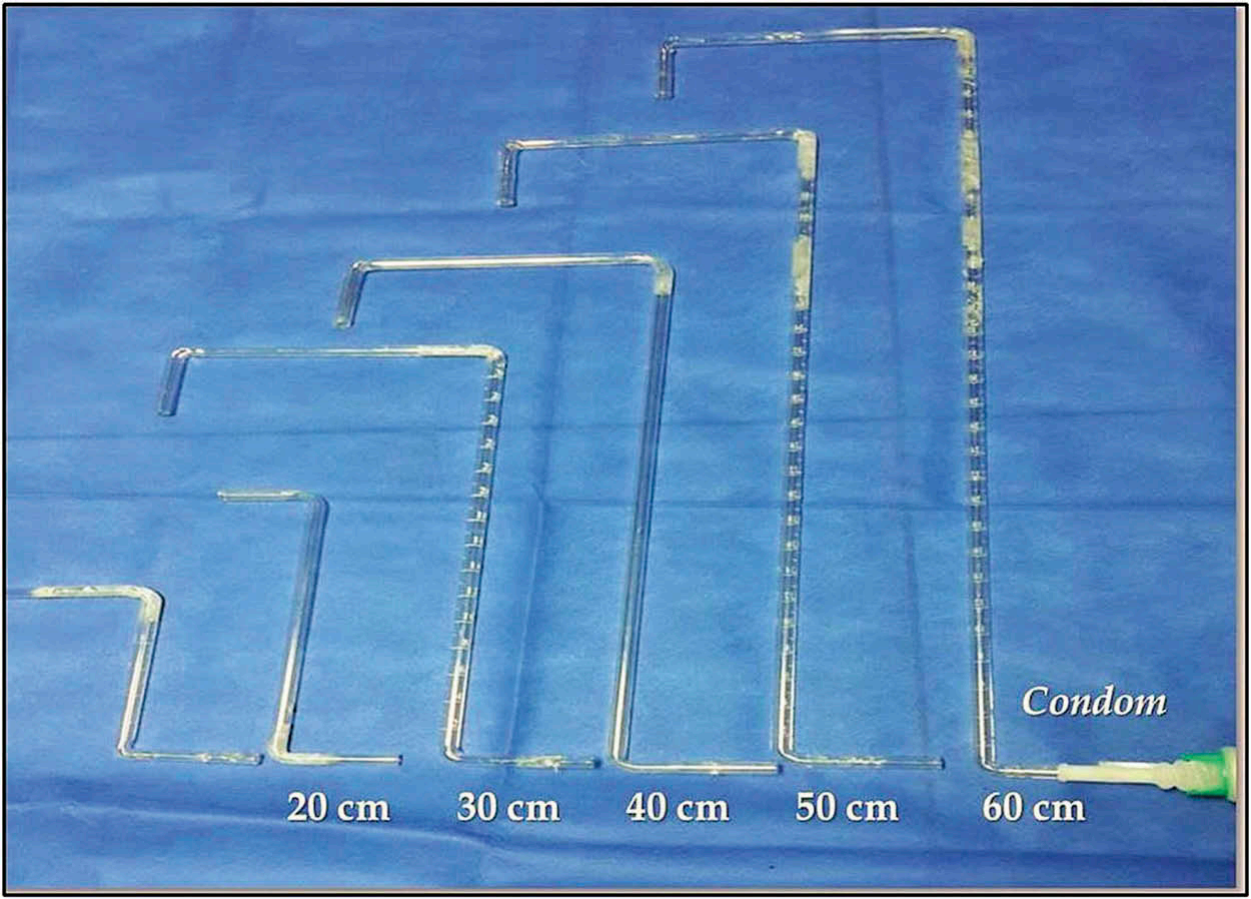
Six different height glass tubes and connected condom catheter.

**Figure 3.**
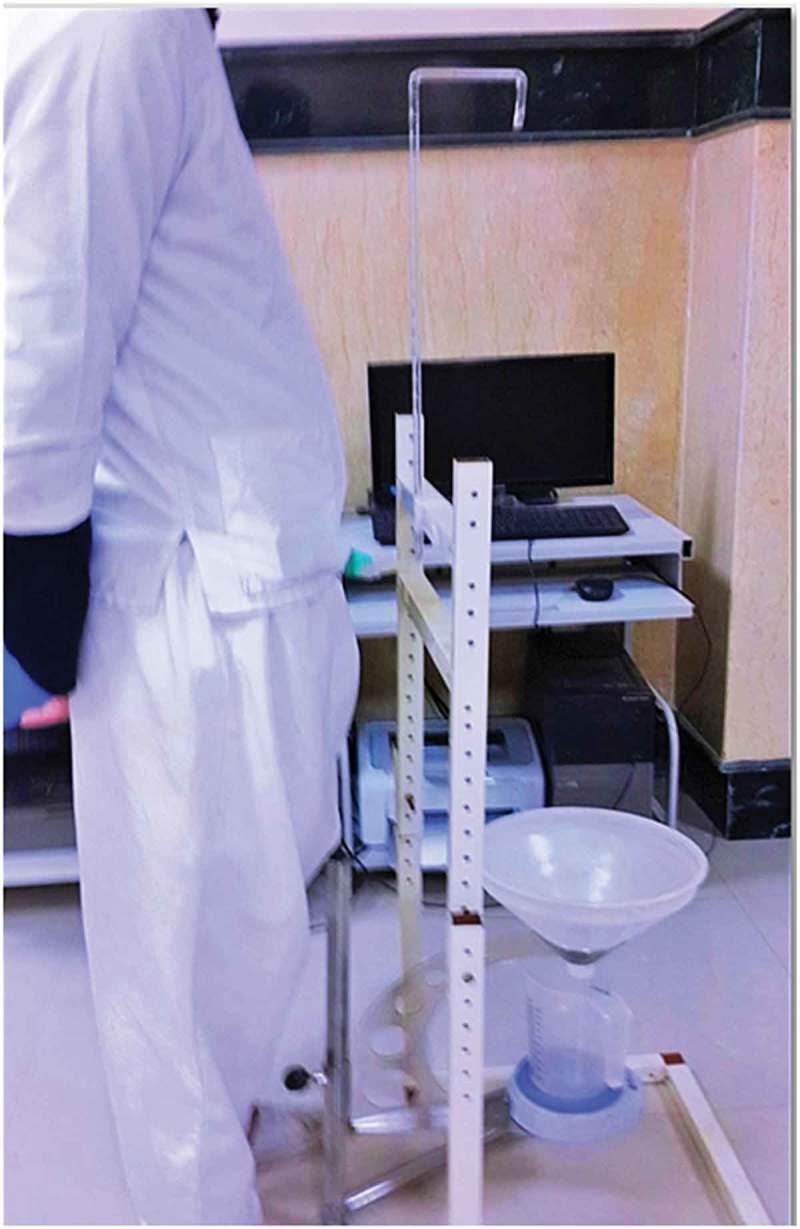
Uroflowmetry through a condom catheter connected to the glass tube.

All the men were asked to undergo an initial pressure–flow study to insure that they were not obstructed and had normal detrusor contractility and a normal P_det_Q_max_ [8,9]. After that, they are asked to come in the next day to the urodynamic laboratory for 6 consecutive days to undergo the novel tube height-resistance test. This non-invasive test starts with free uroflowmetry (zero level) with assessment of PVR using abdominal ultrasonography. Then with a bladder volume ~200 mL, the volunteers were asked to void into a uroflowmeter through a condom catheter fitted to the glans penis and connected to an outflow tube with an 8-F diameter and a specific vertical height above the level of the symphysis pubis (Figures 1–3). The men were advised not to strain during voiding to avoid contribution of abdominal pressure to flow outcome and were closely observed during micturition by the attending physician. If straining was noticed, the test results were cancelled.

The test was started with a 10-cm height and then repeated at increasing heights of 20, 30, 40, 50 and 60 cm on different days to avoid bladder and patient exhaustion. The mean Q_max_ values were compared for each man with his own initial Q_max_ at the initial pressure–flow study, which was considered as the zero level, as the control. The PVR was assessed after each uroflowmetry study. Again, results were compared to the initial recorded PVR (zero level).

A normal Q_max_ was designated as >15 mL/s to avoid a grey zone of equivocal values, whilst a normal PVR was designated as <50 mL (<10% of the normal bladder capacity). We used normal Q_max_ >15 mL/s and normal PVR <50 mL because we found that most researchers and urologists agree with these parameters supported by many published studies [10,11]. These studies showed that the specificity using a threshold for Q_max_ of 15 mL/s to diagnose BOO was 68%, with a positive predictive value (PPV) of 67% and a sensitivity of 82%. On the other hand, using a PVR threshold of 50 mL had a diagnostic accuracy to predict BOO with a PPV of 63% and a negative predictive value (NPV) of 52% [12,13]. No universal agreement regarding both ‘normal’ values could be identified in the literature or in the ICS terminology.

Passing the test successfully means that the Q_max_ and PVR were within the normal range at that height resistance. Receiver operating characteristic (ROC) curve analysis was used to determine the maximum height resistance at which both Q_max_ and PVR remained within the normal range, and this height resistance can therefore be considered as a passing test for that age group of males to identify that they have an adequate voiding reserve.

## Results

All the men were able to continue the study without apparent difficulty. There were no complications, only some difficulty whilst voiding was experienced with the 50- and 60-cm height resistance. Voided volume was >200 mL (range 250–390 mL) in all trial voiding against all levels of height resistance and this ensured that we could rely on the results of the uroflowmetry. The mean (SD) voiding time was 24.1 (3.1) s at 10-cm height resistance and showed a slight increase with further increase in the resistance until reaching 41.1 (3.7) s at 60-cm height resistance (Table 1).

**Table 1.** Subjects’ voiding characteristics at increasing height resistance.

	Height resistance, cm							
Variable, mean (SD)	0	10	20	30	40	50	60	*P*
Q_max_, mL/s	27.6 (1.1)	21.5 (1.3)	21 (1.2)	20.6 (1.1)	20.2 (2.1)	18.3 (1.4)	17.8 (1.5)	<0.001
Q_ave_, mL/s	15.6 (1)	12.8 (0.8)	12.1 (1.1)	12.2 (1.2)	11.8 (0.9)	11.4 (1.2)	10.8 (1.2)	<0.01
Voiding time, sec	24.1 (3.1)	46.3 (4.5)	51.7 (4.9)	52.9 (4.6)	46.4 (5.1)	44.8 (4.1)	41.1 (3.7)	<0.01
Voided volume, ml	338.7 (10.5)	345.5 (15.1)	333.5 (13.3)	359.5 (15.6)	344 (12.5)	291.4 (14.1)	254.3 (11.8)	<0.001
PVR, mL	0	10 (2.5)	16.5 (5.1)	19 (5.6)	21.4 (7.4)	58 (8.1)	65.7 (11.5)	<0.001

The mean (SD) Q_max_ decreased progressively with each increase in height resistance; at zero level it was 27.6 (1.1) mL/s, at the 10-cm level it dropped to 21.5 (1.3) mL/s, then it decreased with each increase of height resistance by smaller values ranging between 0.3 and 1.3 mL/s. At 60-cm height resistance, still 83% of the men had a Q_max_ >15 mL/s [mean (SD) 17.8 (1.5) mL/s]. We noticed that a significant decrease in the mean Q_max_ occurred at the 10-cm resistance tube height (17.9%), whilst further increases in height resistance resulted in slighter decreases in Q_max_ (ranging between 0.7% and 7.2%) (Table 1).

For PVR, it was nil at the zero level and no significant change in PVR was noticed with 10-, 20-, 30- and 40-cm height resistance. PVR increased significantly at the 50-cm height, at a mean (SD) of 58 (8.1) mL, and further increased at 60-cm height resistance to 65.7 (11.5) mL (Table 1).

**Figure 4.**
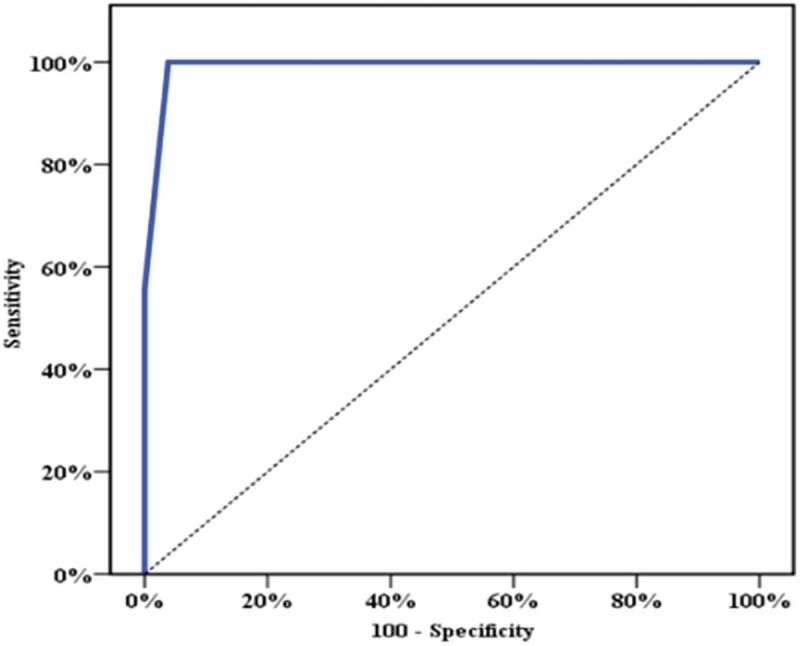
ROC curve for height resistance to diagnose PVR >50 mL.

ROC curve analysis was used for the different levels of height resistance (Figure 4) to determine the height resistance level at which either Q_max_ or PVR, or both changed from normal to abnormal. It was significant for PVR only and showed that the 50-cm height resistance carries the best sensitivity and specificity to be associated with an elevated PVR of >50 mL (100% and 96%, respectively). This was statistically significant (*P* < 0.001), with a 90% PPV and 100% NPV (Table 2).

**Table 2.** Agreement (sensitivity, specificity) for different height resistances to diagnose PVR >50 mL.

AUC	95% CI	*P*	Threshold, cm	Sensitivity, %	Specificity, %	PPV, %	NPV, %
.991*	**0.981–1.0**	<0.001*	10	41.00	57.18	25.0	72.0
			20	55.02	61.09	39.0	70.0
			30	61.00	66.69	45.0	82.0
			40	65.25	76.92	60.0	91.0
			**50**	**100.00**	**96.15**	**90.0**	**100.0**
			60	75.56	100.00	88.0	86.7

AUC, area under the curve.

Bold values show height resistance at which there was a significantly elevated PVR.

So, these results show that the maximum height resistance at which the subjects could pass with a Q_max_ and PVR within normal accepted ranges was 40 cm. This height resistance can be considered as a threshold value for healthy adult males aged <40 years to pass this test successfully with a normal voiding reserve.

## Discussion

All of our body organs are equipped with a special reserve to utilise when facing resistance. This was previously proved in breathing reserve tests and cardiac reserve tests. We think it is time for urologists to devise a non-invasive test to measure voiding reserve. Voiding reserve is a normal physiological process that helps the bladder to overcome increasing outflow resistance keeping the state of compensation in case of BOO. A simple non-invasive way to measure the voiding reserve would be very helpful to assess the ability of the bladder to empty urine efficiently when facing increasing outflow resistance.

Unfortunately, no specific non-invasive method has been developed until now to assess voiding reserve. Most previous studies have been concerned with the role of ultrasonography as a non-invasive test to evaluate bladder muscle function. Amongst the parameters measured by ultrasonography were detrusor wall thickness, intravesical prostatic protrusion, and PVR [14–16]. Other studies have tried to apply graduated outer resistance to urinary flow to assess bladder reserve but the results were not accurate and not reproducible [17,18].

The only available study in the literature regarding any urinary system reserve was the estimation of detrusor muscle reserve published by Yalla and Sullivan [4]. They stated that the detrusor muscle reserve is the difference between P_det_Q_max_ and isovolumetric P_det_. We think it is a pioneer work but it depends on an invasive urodynamic study that needs to be repeated twice to study such a reserve. We tried to create a non-invasive test to avoid the morbidities and difficulties of invasive urodynamics. Instead of occluding the outlet, we forced voiding urine to pass through a height resistance. This concept is well known in physics. If you apply a height resistance in face of flowing liquid you will need more energy to overcome such resistance to maintain the minimum required flow speed.

In our present study, we found that normal young adult males (aged <40 years) can maintain their Q_max_ within the normal range with up to 60-cm height resistance. However, PVR started to rise above the crude accepted normal at 50-cm height resistance. The 40-cm height resistance can therefore be considered a threshold level of resistance that a young healthy adult male should be tested against to estimate his voiding reserve.

The main pitfalls of the test are straining during voiding, leakage of fluid from the condom catheter, patient exhaustion from repeating the test six times and the visual effect of the tube height. We tried to avoid these pitfalls by using a high-quality condom catheter well-fitted to the penis, advising the subjects not to strain, and performing the test on 6 different days to avoid patient exhaustion. The use of a rectal catheter to identify straining was used in some of the patients initially, which we later omitted and were satisfied with the attendant observation only. The visual effect, of watching the tube whilst voiding, was abolished by having a cloth barrier between the patient and the tubes.

We admit that this initial prototype test is still very crude and needs further improvement. We also admit that using glass test tubes is arbitrary and do not carry the same physical properties as the bladder neck and the distensible urethra. This test needs further study regarding reproducibility and standardisation of all variables surrounding the test. However, we are trying to provide urologists with a new and simple non-invasive tool to measure voiding reserve.

We believe that this novel initial prototype test will be helpful for estimating the voiding reserve in males. We are planning to start a new study applying this test to obstructed male patients to evaluate its ability to estimate their voiding reserve non-invasively.

In conclusion, a novel initial prototype of a non-invasive urodynamic test for estimation of voiding reserve is feasible, with no apparent complications or morbidity. A 40-cm height resistance can be considered a reference level that a young adult male should be tested against to estimate his voiding reserve. However, this test still needs to be tested on a larger scale to confirm its validity and reproducibility.
